# Natural products targeting autophagy and apoptosis in NSCLC: a novel therapeutic strategy

**DOI:** 10.3389/fonc.2024.1379698

**Published:** 2024-04-02

**Authors:** Peiyi Qin, Qingchen Li, Qi Zu, Ruxue Dong, Yuanfu Qi

**Affiliations:** ^1^ First Clinical Medical College, Shandong University of Traditional Chinese Medicine, Jinan, China; ^2^ Shandong College of Traditional Chinese Medicine, Yantai, Shandong, China; ^3^ Department of Orthopedics, First Affiliated Hospital of Dalian Medical University, Dalian, China; ^4^ Department of Oncology, Affiliated Hospital of Shandong University of Traditional Chinese Medicine, Jinan, China

**Keywords:** autophagy, apoptosis, crosstalk, therapeutics, natural products, non-small cell lung cancer (NSCLC)

## Abstract

Lung cancer is the leading cause of cancer-related mortality worldwide, with non-small cell lung cancer (NSCLC) being the predominant type. The roles of autophagy and apoptosis in NSCLC present a dual and intricate nature. Additionally, autophagy and apoptosis interconnect through diverse crosstalk molecules. Owing to their multitargeting nature, safety, and efficacy, natural products have emerged as principal sources for NSCLC therapeutic candidates. This review begins with an exploration of the mechanisms of autophagy and apoptosis, proceeds to examine the crosstalk molecules between these processes, and outlines their implications and interactions in NSCLC. Finally, the paper reviews natural products that have been intensively studied against NSCLC targeting autophagy and apoptosis, and summarizes in detail the four most retrieved representative drugs. This paper clarifies good therapeutic effects of natural products in NSCLC by targeting autophagy and apoptosis and aims to promote greater consideration by researchers of natural products as candidates for anti-NSCLC drug discovery.

## 1 Introduction

Lung cancer currently holds the distinction of being the malignancy with the highest rates of mortality globally (1, 2). Approximately 85% of lung cancer cases are classified as non-small cell lung cancer (NSCLC), encompassing adenocarcinoma, squamous carcinoma, large cell carcinoma, among others (3). Studies indicate that the 5-year survival rate for NSCLC in stages III-IV is under 15% (4). Treatment modalities for this cancer typically encompass surgery, radiation, chemotherapy, targeted therapy, and immunotherapy. Surgery combined with platinum-based adjuvant chemotherapy is the first choice for early-stage NSCLC. Yet, it has been reported that over 81% of clinically diagnosed NSCLC patients are ineligible for surgical intervention (5). In cases of advanced NSCLC, it is mostly treated with gemcitabine or docetaxel in combination with platinum drugs. However, the prognosis remains poor due to factors like drug resistance, radio resistance, propensity for recurrence, and pronounced tumor aggressiveness (6–9). Acquired resistance is an urgent problem in cancer treatment, and combination therapies can help to minimize the risk of resistance. Studies have shown that the combination of decitabine and retinoic acid increases sensitivity and therapeutic efficacy in rapidly proliferating and slowly migrating NSCLC (10). The advent of targeted therapies and immunotherapy has provided a glimmer of hope for NSCLC patients. Nevertheless, these treatments are beneficial only for individuals with specific biological markers and are limited by side effects, drug resistance, and high costs (11, 12). Thus, there exists an urgent need to discover more effective, safer, and affordable therapeutic agents.

Inducing tumor cell death is the primary objective in tumor therapy, with the main modes including programmed death (apoptosis, autophagic death), necrosis, pyroptosis, ferroptosis, among others (13). Apoptosis and autophagic cell death are particularly significant in cancer treatment due to their non-inflammatory nature and minimal side effects on the body (14). Autophagy exhibits a tumor-suppressive function by eliminating tumor-inducing molecules in cancer’s early stages. However, in the progression phase, it can support tumor growth by supplying energy to stressed tumor cells under conditions like nutrient scarcity (15). Antitumor drugs can excessively upregulate autophagy-related genes, leading to autophagic death of tumor cells and impeding tumor growth (16). It has also been observed that autophagy can shield tumor cells from radiation and chemotherapy, promoting tolerability and resistance, thereby promoting tumor growth (17). Inhibition of cytoprotective autophagy is considered a promising modality for tumor therapy. In addition to cytoprotective autophagy and cytotoxicity autophagy, David A Gewirtz has proposed two other forms of autophagy, one is a nonprotective form of autophagy, in which the cell apparently performs autophagy-mediated degradation but autophagy inhibition does not result in a significant alteration of chemotherapy or radiation sensitivity and the other is cytostatic autophagy, which results in cell growth inhibition and reduced clonogenicity (16). Therefore, the interplay between autophagy and tumors is bidirectional and intricate, influenced by tissue type, tumor development stage, autophagic activity level, and therapeutic choices (18). Inducing apoptosis in tumor cells has been a focal point in cancer treatment research (19). Moreover, numerous studies reveal complex crosstalk between autophagy and apoptosis. Their interaction and mutual regulation during cancer progression and treatment pose significant challenges in cancer therapy (20). In this context, thorough research into autophagy, apoptosis, and their interplay is vital for effective cancer treatment, including for NSCLC.

Natural products are pharmacological components extracted, isolated, and optimized from nature. Their role in extracting bioactive compounds has demonstrated considerable promise in cancer therapy. Reports indicate that various natural products can influence tumor cells through multiple pathways, including apoptosis, autophagy, cell proliferation, migration/invasion, angiogenesis, and metastasis (21). Statistics show that approximately 47% of antitumor drugs originate from natural compounds (22, 23). Notable anti-cancer drugs like camptothecin, paclitaxel, and vincristine have been effectively utilized in clinical settings, demonstrating significant efficacy (24). The advantages of natural products include low toxicity, high efficiency, multi-targeting capabilities, and affordability. Currently, the therapeutic role of natural products in NSCLC is receiving increasing recognition.

Although autophagy and apoptosis in NSCLC have been extensively explored, a summary of natural products targeting these pathways in NSCLC is lacking. This review initially introduces the mechanisms of autophagy and apoptosis, analyzes the crosstalk molecules between them, and then outlines their roles and interactions in NSCLC. Finally, we reviewed the natural products that target autophagy and apoptosis in NSCLC and summarized the main four of them in detail. This review aims to provide insights for future NSCLC patient treatments.

## 2 Autophagy

### 2.1 Overview of autophagy

The term “*autophagy*” derives from the Greek words, “*auto*” meaning self, and “*phagy*” meaning to eat, defining it as a self-degrading process essential for organ maintenance and energy turnover during injury (25). As an evolutionarily ancient and highly conserved catabolic process, autophagy is classified into macroautophagy, microautophagy, and chaperone-mediated autophagy (CMA), based on the method of substrate delivery to lysosomes (26). Generally, when referring to autophagy, macroautophagy is implied. Macroautophagy serves as a cellular defense mechanism, maintaining homeostasis by degrading and recycling misfolded proteins and damaged organelles (27). Notably, there is substantial evidence suggesting autophagy could be a form of cell death. Excessive autophagy may lead to extensive degradation of cytoplasm and organelles, resulting in type II programmed cell death. Autophagy is further categorized into selective and non-selective types based on the specificity of the degraded substrate. Selective autophagy targets specific components such as intracellular pathogens, damaged organelles, and misfolded protein aggregates, with mitochondrial autophagy being the most extensively studied (28). Non-selective autophagy, in contrast, randomly degrades cytoplasmic substances, including a majority of cytoplasm and organelles. This review will predominantly address macroautophagy, hereafter referred to as autophagy.

### 2.2 The autophagic process

**Figure 1:**
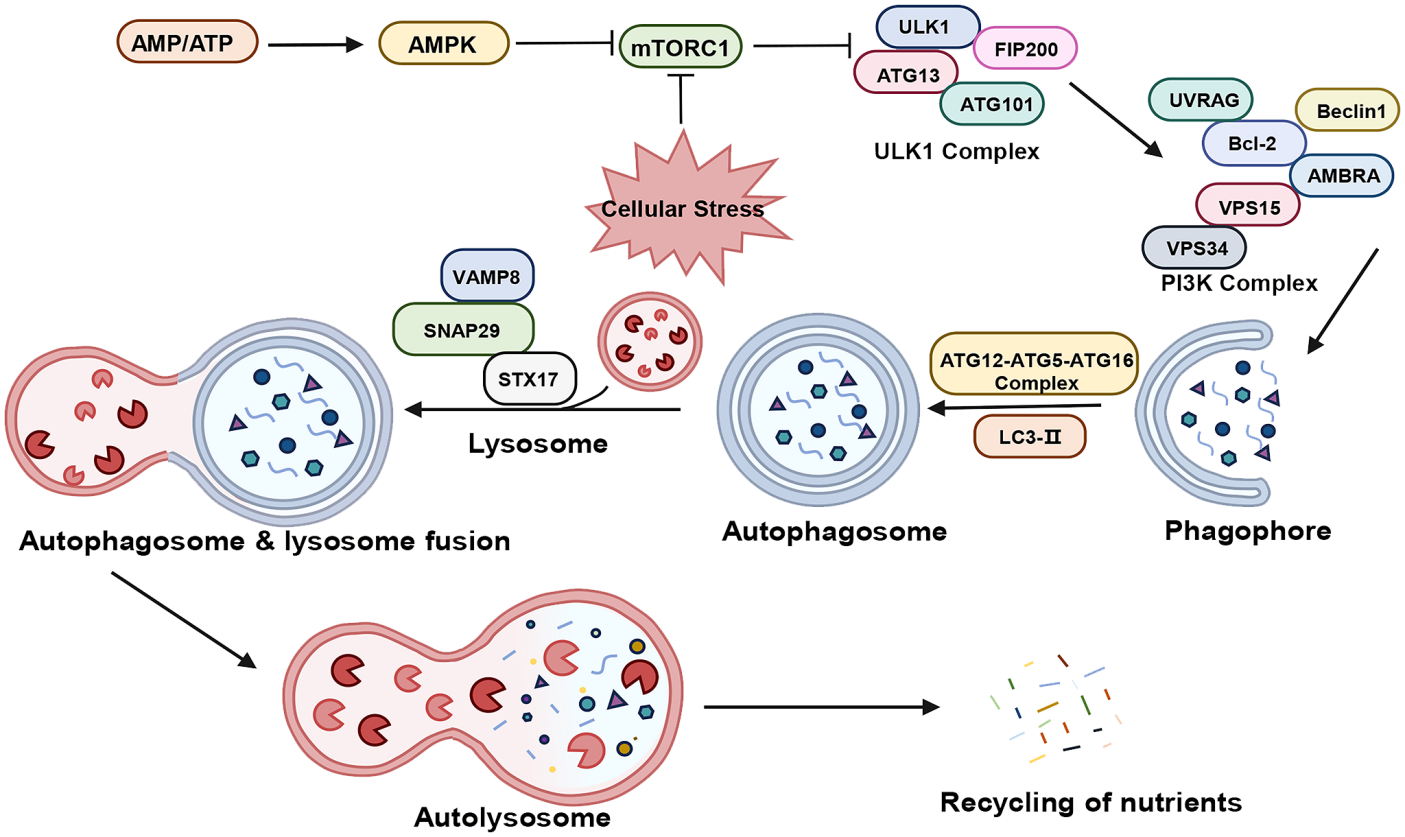
The process of autophagy. “→” represents promotion; “⟂” represents inhibition. AMP, adenosine monophosphate; ATP, adenosine triphosphate; AMPK, AMP-activated protein kinase; mTORC1, mammalian target of rapamycin complex 1; ULKI, Unc-51-like kinase 1; FIP200, FAK family kinase-interacting protein of 200 kDa; ATG, Autophagy Related Protein; UVRAG, ultraviolet radiation resistance-associated gene; Bcl-2, B-Cell Lymphoma 2; AMBRA, autophagy and beclin 1 regulator; Vps34, Vacuolar Protein Sorting 34: Vps15, Vacuolar Protein Sorting 15; PI3K, Phosphatidyl Inositol-3 Kinase; LC3, Light Chain Kinase 3; STX17, syntaxin 17; SNAP29, Synaptosomal-associated protein 29; VAMP8, vesicle-associated membrane protein 8.

Macroautophagy, hereafter referred to as autophagy, encompasses a series of signaling cascades involving various autophagy-associated genes, proteins, and compounds (29–31). As illustrated in **Figure 1**, this process includes the induction, nucleation, elongation, maturation, and closure of phagosomes, ultimately leading to autophagosome formation and their fusion with lysosomes.

In the physiological state, the cell maintains a very low level of basal autophagy. At this time, there is sufficient intracellular energy, Mammalian target of rapamycin complex 1 (mTORC1) is in an activated state and phosphorylates Autophagy Related Protein (ATG) 13, thus inhibiting cellular autophagy. Under stress such as hypoxia, the reduction in ATP levels activates AMP-activated protein kinase (AMPK), which phosphorylates tuberous sclerosis complex 2 (TSC2) to inhibit mTORC1 activity (32, 33). the activity of mTORC1 is inhibited, the phosphorylation level of ATG13 decreases, and the dephosphorylated ATG13 forms a complex with Unc-51-like kinase 1 (ULK1) and interacts with FAK family kinase-interacting protein of 200 kDa (FIP200) to produce the ATG13-ULK1-FIP200 complex, and induces downstream autophagosome nucleation and elongation (34–36). The nucleation process is closely related to the Vacuolar Protein Sorting 34 (Vps34) - Beclin-1 complex, which also contains Vacuolar Protein Sorting 15 (Vps15), and together they act in the nucleation of membrane vesicles and mediate the formation of pre-autophagosomal structure (PAS) (37, 38). The extension of mammalian autophagosome mainly depends on two ubiquitination-like systems: the binding process of ATG12 and the modification process of Light Chain Kinase 3 (LC3) (39, 40). Binding process of ATG12: ATG12 is first activated by ATG7, then transporter and bound to ATG5 via ATG10, and then bound to ATG16, generating a multibody complex of ATG12-ATG5-ATG16. This complex is localized on the outer membrane surface of the preautophagosome structure and participates in the expansion of the outer membrane of the preautophagosome (41). The process of LC3 modification: after the formation of LC3 precursor, it is processed into cytoplasmic soluble LC3-I by ATG4, and then covalently linked to phosphatidylethanolamine (PE) to become lipid-soluble LC3-PE (LC3-II) by ATG7 and ATG3, and participates in the membrane elongation. LC3-II is able to bind to newly formed membrane until the formation of autophagic lysosome formation. Therefore, LC3-II is commonly used as a marker for autophagy formation and is an important multi-signaling regulatory protein localized on the membrane of autophagic vesicles (42, 43). In addition, p62/SQSM1 is involved in autophagosome formation and promotes translocation of the mTORC1 complex to the lysosomal surface, where it is selectively encapsulated into the autophagosome and subsequently degraded by protein hydrolases in autophagic lysosomes (44). Thus, the expression of p62/SQSM1 protein showed a negative correlation with autophagic activity. Subsequently, the Soluble n-Ethylmaleimide Sensitive Factor Attachment Protein Receptor (SNARE) protein family orchestrates the binding of the autophagosome with the lysosome, forming the autophagolysosome (45). Lastly, lysosomal acid hydrolases decompose the cargoes within the autophagosome, with the breakdown products reabsorbed for recycling.

### 2.3 The four faces of autophagy in NSCLC

In early NSCLC, autophagy suppresses tumor initiation by removing damaged organelles and misfolded proteins, thus preserving cell homeostasis. Inhibition of cellular autophagy promotes tumor cell growth, showing that cellular autophagy inhibits tumorigenesis (46). However, in mid-to-late stages, autophagy is generally considered to facilitate NSCLC progression by supplying nutrients and energy to cancer cells, enabling their adaptation to various stress states. At this stage, elevated autophagy levels are positively associated with increased cancer cell invasiveness, advanced tumor stages, and reduced survival (47). In addition to the cytoprotective (cell survival) and cytotoxic (cell death) roles of autophagy described above. Bai et al. suggested that autophagy also exists in cytostatic (cell growth arrest) and nonprotective (no contribution to cell death or survival) forms in tumor cells (48).

The four faces in the process of tumor therapy: Firstly, Tumor cell autophagy protects tumor cells from damage caused by chemotherapy and radiotherapy and promotes their metastasis, invasion and drug resistance. Studies reveal that autophagy contributes to chemotherapy resistance in NSCLC, whereas autophagy inhibition can counteract tumor resistance and progression (49). Li et al. demonstrated that protein DICER, through its interaction with let-7i-5p, activates autophagy in NSCLC, thereby promoting cisplatin resistance (50). Yuqing Chen et al. confirmed that overexpression of miR-142-3p enhances chemotherapy sensitivity in NSCLC by suppressing autophagy (51). Secondly, autophagy can in turn induce programmed apoptosis or autophagic death in tumor cells because of the effects of antitumor drugs. PFAP, a newly identified protein from Pleurotus ferulae lanzi, a Chinese medicinal herb, has shown therapeutic potential in NSCLC. *In vivo* and *in vitro* studies demonstrated that PFAP induces autophagy in A549 cells via the AMPK/mTOR pathway, significantly reducing the growth of xenograft tumors in nude mice (52). Similarly, Paris saponin VII (PSVII), extracted from Trillium tschonoskii Maxim and studied by Xiang et al., activates autophagy through the AMPK/mTOR signaling pathway, inhibiting the proliferation of NSCLC cells and exhibiting anti-cancer effects (53). Thirdly, Sharma et al. demonstrated a novel cytostatic form of autophagy induced by vitamin D and its analog, EB 1089, which sensitizes NSCLC cells to radiotherapy. This effect is potentially mediated by VDR, TP53, and AMPK, contributing to an enhanced response to radiation in NSCLC by 1,25-D3 and EB1089 (54). Fourthly, Patel et al. confirmed that inhibition of autophagy to overcome radiotherapy resistance might only be effective if autophagy serves a cytoprotective role. In NSCLC, autophagy can manifest as nonprotective, and p53 does not reliably indicate autophagy’s functional status, suggesting that the inclusion of autophagy inhibitors in treatments requires careful screening (55).

## 3 Apoptosis

### 3.1 Overview of apoptosis

The term “apoptosis” originates from the Greek “apó” (from) and “ptósi” (fall), signifying “to fall off” as leaves from a tree or petals from a flower. Kerr et al. first described the apoptotic morphology of cells in the 1970s (56). Apoptosis, also known as type I programmed cell death, is an active, physiological cellular death process, driven by internal genetic mechanisms under certain physiological or pathological conditions. Triggered by intracellular and/or extracellular signals, apoptosis involves a sequence of morphological changes, such as cell shrinkage, chromatin condensation, nuclear fragmentation, and plasma membrane blistering, culminating in apoptotic body formation (56, 57). Apoptosis is crucial in maintaining internal environmental stability and various systemic physiological functions (58), Abnormal apoptosis is implicated in the development of tumors, neurodegenerative diseases, cardiovascular disorders, and autoimmune diseases (59).

### 3.2 The apoptotic cascades

**Figure 2:**
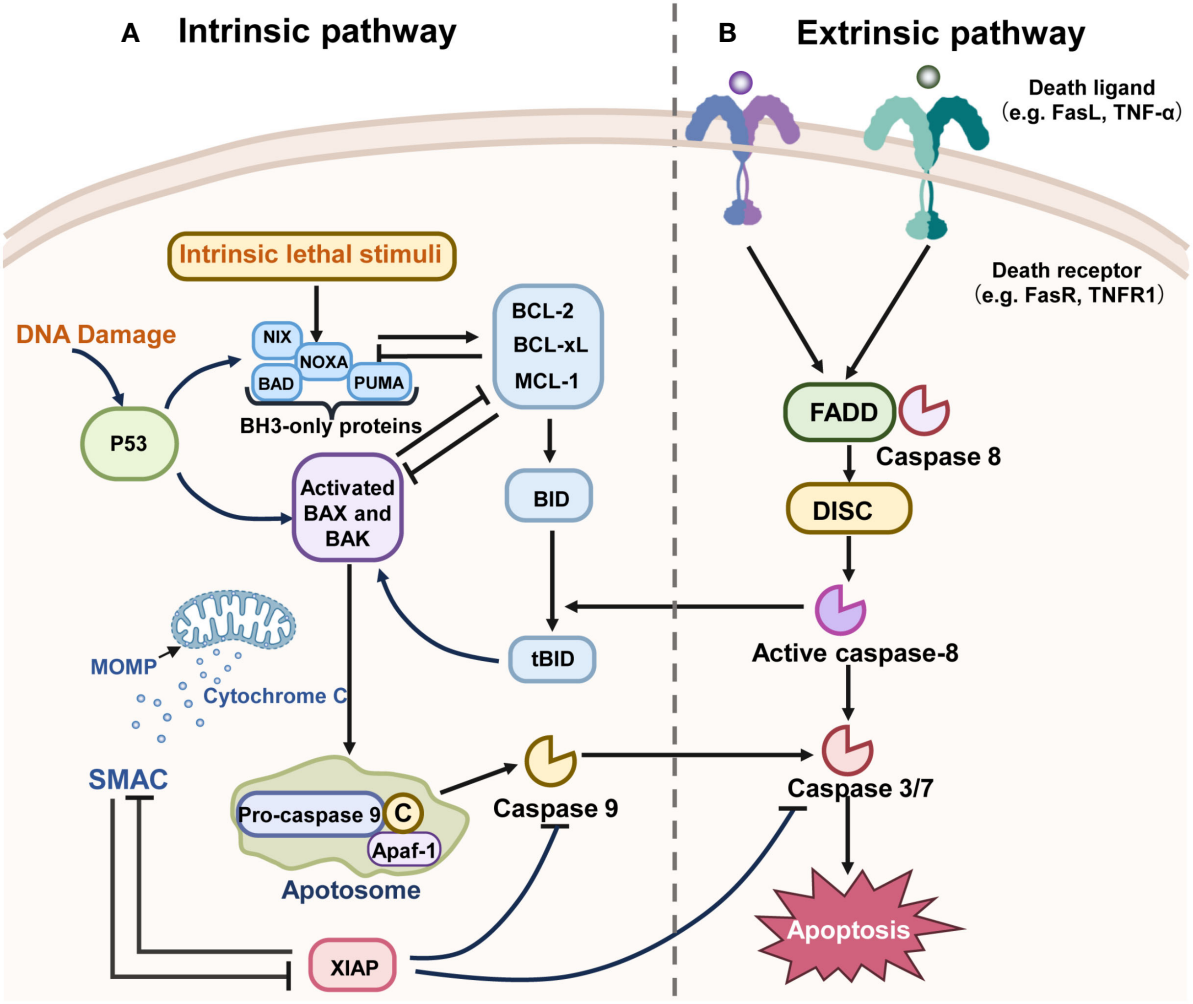
The mechanisms of the two main pathways of apoptosis. “→” represents promotion; “⟂” represents inhibition. **(A)** The intrinsic pathway of apoptosis is regulated by the BCL-2 family of proteins and can be initiated by various stimuli. For example, BH3-only proteins such as PUMA and NOXA are activated directly through transcriptional regulation by P53. These BH3-only proteins activate the pro-apoptotic proteins Bax or Bak, inhibiting the anti-apoptotic proteins like Bcl-2, BCL-XL, and MCL-1. The activation of Bax or Bak leads to the initiation of MOMP, which in turn activates the cascade, culminating in cell apoptosis. X-linked inhibitor of apoptosis (XIAP), a member of the Inhibitor of Apoptosis Protein (IAP) family that is often overexpressed in malignancies, functions to inhibit caspase activation and can be inactivated by SMAC. **(B)** The death receptor pathway is initiated by the activation of caspase-8, Fas, TNF, and TNF receptors, which recruit death domain proteins such as FADD and TRADD, as well as caspases, to form death-inducing signaling complexes (DISC). Other downstream molecules have been activated by DISC and lead to apoptosis.

As illustrated in **Figure 2**, the apoptotic process encompasses interactions among various proteins, signal transducers, and pathway cascades (60–62).

The extrinsic apoptotic pathway, or death receptor pathway, is triggered by external stimuli. It induces apoptosis through interactions between death ligands and receptors. Death receptors, belonging to the tumor necrosis factor family, include Tumor Necrosis Factor Receptor Type 1 (TNFR1) and Fas receptor. In this pathway, binding of a death receptor to specific ligands like Tumor Necrosis Factor (TNF) or Tumor Necrosis Factor-Related Apoptosis Inducing Ligand (TRAIL) transmits the death signal into the cytoplasm, activating the intracellular apoptotic pathway (63–65). The interaction of the death receptor and ligand recruits TNFR1-Associated Death Domain (TRADD), Fas-Associated Death Domain Protein (FADD), Tumor Necrosis Factor Receptor-Associated Factor 2 (TRAF2), and Caspase-8, forming the death-inducing signaling complex (DISC). DISC initiates Protease-8 activation, which in turn triggers downstream effector proteins, thereby inducing apoptosis (66, 67).

The intrinsic apoptotic pathway, also known as the mitochondrial pathway, is initiated by mitochondrial outer membrane permeabilization (MOMP) mediated by the B-Cell Lymphoma 2 (Bcl-2) protein family under conditions like DNA damage, energy starvation, and hypoxia (67). The Bcl-2 family, pivotal in regulating endogenous apoptosis, is categorized based on the number of Bcl-2 homology (BH) domains (68). Pro-survival proteins contain BH1-BH4 domains (e.g., Bcl-XL, Bcl-2, Mcl-1), while pro-apoptotic proteins possess BH1-BH3 domains (e.g., Bax, Bak) (69). BH3 proteins detect DNA damage and signal apoptosis. Upon their migration to the mitochondrial membrane, they activate pro-apoptotic proteins Bax or Bak, or inhibit anti-apoptotic proteins like Bcl-2 and Myeloid Cell Leukemia 1 (Mcl-1) (70). Activation of pro-apoptotic proteins in mitochondria leads to MOMP, followed by cytochrome c release into the cytoplasm. Cytochrome c binds with Apoptotic Protease Activating Factor 1 (APAF-1) to form an apoptotic complex, activating Caspase 9’s precursor, which in turn activates Caspase 3 and Caspase 7, inducing a caspase cascade and triggering cell apoptosis (71). In summary, intrinsic apoptosis involves three key stages (1): Bcl-2 family protein interactions (2), Bax/Bak-mediated MOMP, (3) activation of apoptotic caspases (72). Both mitochondrial and death receptor pathways rely on caspase activation (73).

### 3.3 The roles of apoptosis in NSCLC

The emergence of cancer from normal tissue cells involves genomic instability and/or inflammation. One natural defense mechanism cancer cell employs to promote their growth and survival is the inhibition of apoptosis (74). Therefore, inducing apoptosis has been identified as a potential cancer treatment strategy (56).

In NSCLC treatment, numerous drugs work by triggering apoptosis in cancer cells. It is reported that Erufosine, a novel alkylphosphocholine, exhibits anti-tumor effects on NSCLC cells by inducing apoptosis and cell cycle arrest (75). Natural products also play a role in NSCLC treatment by modulating apoptosis. Lobocrassin B, a membrane-type compound from Chassophyllum soft coral, has shown potential as an anti-lung cancer agent. It increases levels of Bax, cleaved Caspase-3, -8 and-9, and decreases Bcl-2 in CL1-5 and H520 cells, effectively inducing lung cancer cell death through the mitochondria-dependent apoptosis pathway (76). Isoflavones (Biochanin A), phytoestrogens found in legumes, have been observed to induce lung cancer cell cycle arrest and apoptosis in A549 and 95D cells by regulating cell cycle-related protein expression and regulating the Bcl-2 and Caspase-3 pathways (77). Similarly, Lotus leaf flavone upregulates Caspase-3, -9, and Bax, and downregulates Bcl-2 expression in A549 cells, inhibiting cell proliferation and inducing apoptosis without harming normal cells (78). Additionally, the flavonoid vitexin induces apoptosis in A549 cells, downregulates the Bcl-2/Bax ratio, and enhances caspase-3 expression, promoting apoptosis (79).

Paradoxically, Kerr et al. observed that “spontaneous and persistent cell death is an inherent characteristic of malignant tumors”. Induction of apoptosis is often considered an important way to prevent and treat cancer. However, apoptosis may also lead to unexpected results and may even promote cancer. In the apoptotic pathway, cleavage of cysteine asparaginase activates calcium-independent phospholipase A2 (iPLA2), which produces prostaglandin E2 (PGE2) that enhances proliferation. Also in this process, apoptosis provides support for proliferating cells by removing tumor cells that respond to treatment, leading to tumor regeneration and chemoresistance (80, 81). These findings suggest a dual role for apoptosis in tumor regulation, highlighting that targeting apoptosis alone in treatment may inadvertently promote cancer progression or recurrence. Therefore, in cancer therapy, particularly NSCLC, it is crucial to consider other intracellular regulatory mechanisms. A comprehensive approach that includes differentiation and apoptosis could effectively regulate NSCLC.

## 4 Crosstalk between autophagy and apoptosis

### 4.1 Linking autophagy and apoptosis

Autophagy and apoptosis, two highly conserved evolutionary processes, collaborate to maintain cell homeostasis under stress. Despite differing physiological mechanisms, they are closely interconnected, sharing several key regulatory factors (82). The interplay between autophagy and apoptosis in NSCLC is primarily governed by Beclin-1, ATG5-ATG12, and caspase family proteins. This review focuses on a few regulatory factors in their crosstalk, with additional targets outlined in **Table 1**.

**Table 1:** List of target biomarkers regulating the autophagy-apoptosis crosstalk.

Biomarkers	Acting genes/locations	Roles in autophagy and apoptosis	Reference
Calpain	ATG5, P62	Calpain induced apoptosis and inhibited autophagy by cutting ATG5.	(83)
SQSTM1/P62	mTOR, Nrf2, Caspase-8	P62 is involved in the formation of autophagosomes and promotes the translocation of the mTORC1 complex to the lysosomal surface, thereby inhibiting autophagy. P62 promotes the activation of Caspase 8 and the downstream effector Caspase 8 and induces apoptosis.	(44, 84, 85)
FADD	ATG5	FADD induced autophagy through its interaction with ATG5, and activated Caspase-8 to induce apoptosis.	(86)
DAPK1	Vps34, TNF-α, INF-γ	DAPK1 induces autophagosome formation by regulating Vps34, and is widely involved in the positive induction of apoptosis by TNF-α, IFN-γ, Fas and other pathways.	(87–89)
ROS	HIF-1, P53, MAPK	ROS may aggravate cell damage and induce autophagy through oxidative stress. At the same time, apoptosis was induced through mitochondrial pathway.	(90, 91)
FLIP	ATG3-LC3, FADD, TRAIL	FLIP is an anti-apoptotic protein involved in death receptor mediated apoptosis and also blocks autophagy by preventing ATG3 binding to LC3.	(92, 93)
PI3K/Akt pathway	mTOR	PI3K/Akt pathway inhibits mTOR and promotes autophagy. It also negatively regulates apoptosis.	(94, 95)
ATG3-ATG12	Mitochondria	The formation of the ATG3-ATG12 complex significantly reduces the selective autophagy process and mediates the inhibition of apoptosis in the mitochondrial pathway.	(96, 97)
AMBRA1	Beclin-1, ATG12, Calpain	AMBRA1 interacts with Beclin-1 to promote the formation of autophagosomes. In mitochondria, the interaction of Ambra1 with Bcl-2 regulates Beclin-1-dependent apoptosis Cells are sensitive to apoptosis when AMBRA1 is down-regulated.	(98–100)
Caspase 8	ATG3, ATG5, Beclin-1	Caspase 8 is a promoter in the exogenous apoptotic pathway, which can directly induce apoptosis and inhibit autophagy by acting with ATG3, ATG5 and Beclin-1.	(101)
Bim	Beclin-1, BCL-2	BIM recruits Beclin-1 to microcells to inhibit autophagy and can also inhibit BCL-2 to promote apoptosis.	(102–104)
HIF-2α	Fas	HIF-2α enhances Fas expression to promote apoptosis and regulates cellular autophagy.	(105)
UVRAG	ERPHS, Bax	UVRAG enhances the assembly of endoplasmic reticulum autophagy initiation sites (ERPHS) and promotes endoplasmic reticulum autophagy, and also binds to Bax and induces cell apoptosis.	(106–108)

ATG, Autophagy Related Protein; P62/SQSTM1, Sequestosome-1; mTOR, Mammalian target of rapamycin; FADD, Fas-Associated Death Domain Protein; Nrf2, NF-E2-related factor 2; Vps34, Vacuolar Protein Sorting 34; DAPK1, death associated protein kinase1; HIF-1, hypoxia inducible factor-1; FLIP, FLICE inhibitory protein; PI3K, Phosphatidyl Inositol-3 Kinase; AMBRA1, autophagy and beclin 1 regulator 1; TNF-α, tumor necrosis factor-α; INF-γ, interferon -γ; ROS, reactive oxygen species; MAPK, mitogen-activated protein kinase; LC3, Light Chain Kinase 3; TRAIL, Tumor Necrosis Factor-Related Apoptosis Inducing Ligand; Bcl-2, B-Cell Lymphoma 2; BIM, Bcl-2 interacting mediator of cell death; HIF-2α, Hypoxia Inducible Factor 2 Alpha; UVRAG, UV Radiation Resistance-Associated Gene Protein.

#### 4.1.1 Beclin-1 and Bcl-2

The Beclin-1 and Bcl-2 interaction plays a crucial role in the autophagy-apoptosis crosstalk (109). Beclin-1, the mammalian homologue of yeast ATG6/Vacuolar Protein Sorting 30 (Vps30) and also known as the BECN1 gene, is recognized as a tumor suppressor gene. Bcl-2 family proteins, including anti-apoptotic proteins (Bcl-2, Bcl-xl, etc.) and pro-apoptotic proteins (BH3-only proteins, Bax, Bak, etc.), regulate MOMP in apoptosis’ intrinsic pathway (110). Beclin-1, pivotal in autophagy nucleation, is essential for autophagy regulation (111). As a central protein, Beclin-1 assembles cofactors to form the BECN1 (beclin 1) - phosphatidylinositol 3-kinase catalytic subunit type 3 (PIK3C3) - phosphatidylinositol 3-kinase regulatory subunit 4 (PIK3R4) complex, initiating the autophagy protein cascade, and holds dual roles in NSCLC autophagy regulation (112). Beclin-1, also a BH3-only protein, regulates both autophagy and apoptosis by binding with the Bcl-2 anti-apoptotic protein. Bcl-2, sharing the BH3 domain with Beclin-1, can bind to Beclin-1 via this domain, inhibiting autophagy when complexed together. Autophagy is induced when Bax competitively binds Bcl-2, dissociating Beclin-1 (101, 113, 114). Additionally, stressors like nutrient deficiency can activate c-Jun N-terminal Kinase 1 (JNK1), leading to Bcl-2 phosphorylation and subsequent autophagy promotion (115).

#### 4.1.2 ATG

Autophagy involves various conserved autophagy-associated genes, with ATG5 and ATG12 being crucial not only for autophagosome formation but also in apoptosis (116). ATG5, instrumental in autophagic vacuole membrane elongation, combines with ATG12 to form the ATG12-ATG5 conjugate (116). In apoptotic cells, Calpain cleaves ATG5, producing truncated fragments that migrate to mitochondria and interact with Bcl-XL, inducing cytochrome C release and triggering apoptosis (83). Rubinstein et al. identified ATG12 as ATG5’s binding partner, contributing to autophagosome extension. ATG12 also plays a role in apoptosis, possibly by binding and inactivating anti-apoptotic Bcl-2 and Mcl-1 proteins (117). Additionally, ATG4D is a key mediator in autophagy and apoptosis crosstalk. ATG4, a cysteine protease originating from yeast, facilitates the attachment of ATG8 to phagocytic vesicles by cleaving ATG8 ubiquitin-like proteins’ C-terminus. Research indicates that Caspase-3 cleaves ATG4D, prompting mitochondria-targeted apoptosis (118). These findings suggest that ATG5, ATG12, and ATG4 may act as critical switch proteins between autophagy and apoptosis.

#### 4.1.3 Caspases

Caspases, a group of cysteine proteases, are primary facilitators of apoptosis and also influence the crosstalk between autophagy and apoptosis (119, 120). Apoptotic caspases are categorized into initiators (Caspase 2, -8, -9, and -10) and effectors (Caspase -3, -6, and -7) (121, 122). These enzymes can degrade autophagy proteins, thereby inhibiting autophagy and ending cellular self-protection. ATG3, crucial in LC3 lipidation and autophagosome formation, can be cleaved by Caspase-8, impeding autophagy and promoting apoptosis (98). Additionally, activated caspases can convert autophagy protein fragments into pro-apoptotic fragments, triggering apoptosis (120). Calpain, a widely conserved family of calcium-sensitive cysteine proteases present in cytoplasm and mitochondria (123), plays a significant role. As previously mentioned, Calpain cleaves ATG5 to generate a fragment that translocate to mitochondria and promotes cytochrome C release, sensitizing cells to apoptosis. Mitochondrial autophagy (mitophagy) is the gradual accumulation of mitochondrial DNA mutations in response to stresses such as reactive oxygen species (ROS) stress, resulting in a decrease in intracellular mitochondrial membrane potential and damage to depolarization, and ultimately leading to cell death (124, 125). Calpain also degrades ATG5, thereby inhibiting mitochondrial autophagy in selective autophagy processes (83). Caspase-3, a critical terminal enzyme in apoptosis, also affects autophagy. It inhibits autophagy by cleaving Beclin-1, and the cleaved Beclin-1 moves to mitochondria, facilitating cytochrome C release from the mitochondrial outer membrane, which promotes apoptosis (126). Initiator Caspase-9, part of the intrinsic apoptosis pathway, can interact with ATG7 to initiate autophagy (92). Thus, caspases are integral in regulating both apoptosis and autophagy.

#### 4.1.4 P53

The transcription factor P53, encoded by the TP53 gene, is a crucial tumor suppressor involved in cell cycle regulation, DNA repair, and apoptosis (127). Under normal, unstressed conditions, P53 is maintained at low levels, marked for degradation by E3 ubiquitin ligase murine double minute 2 (MDM2) (128). However, in response to stressors like DNA damage, oxidative stress, or nutrient deficiency, P53 activates, leading to cell cycle arrest, apoptosis, or senescence (129). Although wild-type TP53 can repair DNA damage and induce tumor cell apoptosis, mutations in the TP53 gene are present in over half of all cancer cases (130). P53 is a well-known apoptosis inducer, promoting transcription of pro-apoptotic genes Bax, and suppressing anti-apoptotic gene Bcl-2, thus triggering apoptosis (131). Beyond apoptosis, P53 also regulates autophagy. Cytoplasmic P53 can inhibit autophagy by interacting with autophagy protein FIP200, thereby competitively impeding autophagy (121, 132). As a transcription factor, P53 can enhance autophagy in the nucleus by activating upstream regulatory factors of mTOR. This involves two primary pathways: the first regulating autophagy via damage factor Damage-Regulated Autophagy Modulator (DRAM), and the second through AMP-activated protein kinase (AMPK) activation. Nuclear P53 transcriptionally activates the DRAM to boost autophagy (133). Additionally, nuclear P53 can activate AMPK, tuberous sclerosis complex 1 (TSC1) and TSC2, subsequently inhibiting mTOR and promoting autophagy (134). In conclusion, P53 plays a vital role in modulating the interplay between autophagy and apoptosis.

### 4.2 Main effects of interaction between autophagy and apoptosis in NSCLC

Under normal conditions, autophagy typically precedes apoptosis, occurring in response to various cellular stresses (135). Ideally, both autophagy and apoptosis exert anti-tumor effects: autophagy by eliminating tumor cells and apoptosis by preventing their survival. However, increasing evidence suggests that autophagy can enhance cancer cell survival and confer chemotherapy resistance in certain cancers (136). Thus, autophagy’s role in cancer development is dual: on one hand, it can inhibit apoptosis and promote tumor cell growth through protective autophagy; on the other, excessive autophagy leads to type II programmed cell death, or “ACD”. “ACD” is characterized by abundant autophagosomes and autophagolysosomes in the cytoplasm, extensive cytoplasmic degradation, but an intact nucleus, typically not reliant on Caspase family activity (137, 138). The concept of autophagy-related cell death is debated, as autophagy-mediated apoptosis and a distinct cell death mechanism (independent of apoptosis and necrosis) are all referred to as “ACD” (139). Whether autophagy promotes or inhibits apoptosis depends on the cell type, stress nature, and stress duration (140). The interaction between autophagy and apoptosis in NSCLC is complex, with their relationship categorized into three main types: promotional, synergistic, and antagonistic.

#### 4.2.1 Promoting effect

Over-activated autophagy, leading to extensive degradation of cellular components by lysosomes, can trigger cell death (141). Autophagy-induced apoptosis can occur directly or via shared regulatory factors (142). TNF-α, a pro-inflammatory cytokine primarily produced by macrophages and monocytes, is implicated in normal inflammatory responses and immune cell regulation. In human neutrophils, autophagy-mediated TNF-α can induce early apoptosis (143), indicating that autophagy might prompt apoptosis through common regulators like TNF-α. Additionally, studies have shown that inhibiting autophagy with 3-MA also reduces cell apoptosis (144). Bruceine D (BD), a steroidal compound, induces apoptosis and autophagy in A549 and NCI-H292 cells through reactive oxygen species (ROS) production and mitogen-activated protein kinase (MAPK) pathway regulation; here, autophagy inhibition diminishes BD-induced apoptosis (145).

#### 4.2.2 Synergy effect

Autophagy and apoptosis can have a synergistic effect, either occurring simultaneously or compensating for each other’s deficiency. For instance, during radiotherapy under nutrient deprivation, increased autophagy levels supply ATP to cells while releasing apoptotic signals, leading to cancer cell apoptosis (146). In cells lacking apoptosis ability, endoplasmic reticulum stress-induced apoptosis is suppressed, but persistent autophagy can cause oxidative damage and ultimately induce apoptosis (147). transmembrane protein 100 (TMEM100), a potential tumor suppressor, enhances autophagy and synergistically induces apoptosis in NSCLC cells (148). Licarin A, extracted from nutmeg, activates a synergistic effect of autophagy and apoptosis, culminating in NSCLC cell death (149). It was demonstrated that the Heat shock protein 90 (HSP90) inhibitor DPB could inhibit the growth of A549 cells by co-inducing apoptosis and autophagy to exert anti-NSCLC effects (150). The above studies suggest that apoptosis and autophagy have complementary synergistic roles and work together to promote cell death.

#### 4.2.3 Antagonism effect

Under normal physiological conditions, autophagy primarily serves as a cell protection mechanism to avert cell death (151). In cancer, rapid, exponential growth often leads to tumor cells experiencing starvation and hypoxia. Autophagy, in response to genomic instability and/or inflammatory reactions, can adapt tumor cells to these stressors by degrading mitochondria. Cellular degradation of mitochondria through mitochondrial autophagy reduces ROS by eliminating depolarized mitochondria for cytoprotective purposes. Mitochondrial autophagy prevents apoptosis by reducing MOMP and decreasing the release of cytochrome C (152). This protective autophagy, however, can contribute to chemotherapy resistance, impeding cancer treatment (17). Chloroquine, a widely used autophagy inhibitor, has been shown to inhibit bevacizumab-induced autophagy, promoting apoptosis and inhibiting tumor cell proliferation (153). Research indicates that inhibiting autophagy can enhance cisplatin-induced apoptosis (154). Almonertinib, a third-generation epidermal growth factor receptor (EGFR) -targeting drug, induces both apoptosis and autophagy in NSCLC cells by promoting ROS production. The autophagy induced is cytoprotective; thus, blocking autophagy promotes apoptosis (155). The Pinocchio protein has been found to inhibit autophagy, thereby promoting apoptosis and inhibiting proliferation in A549 cells, exhibiting anti-NSCLC effects (156). Additionally, the expression of the brain-expressed X-linked 2 (BEX2) gene promote mitochondrial autophagy in NSCLC cells and inhibits cell apoptosis (157). The antagonism between autophagy and apoptosis is common in cancer, often leading to treatment-resistant cancers. Hence, targeting autophagy inhibition to enhance tumor cell apoptosis is considered an effective anticancer therapy strategy.

## 5 Effects of four natural products regulating autophagy and apoptosis on NSCLC

Extensive research indicates that various natural products can therapeutically influence NSCLC by modulating autophagy and apoptosis (21, 158, 159). Natural products such as camptothecin, vincristine and paclitaxel have been successfully used in clinical applications, and the search for active ingredients with anti-tumor activity and low toxicity from natural products has become a hot research topic for researchers in recent years. The mechanisms of anti-NSCLC activity of natural products from different sources in recent years have focused on inducing apoptosis, blocking the cell cycle, regulating cellular autophagy, promoting histone acetylation, inhibiting hypoxia-induced processes, increasing the level of ROS, inhibiting invasion and metastasis, and reversing drug resistance (160). In this article, we review the natural products that are currently being studied in NSCLC therapy with a focus on the regulation of autophagy and apoptosis. Through searching we found that dihydroartemisinin, curcumin, baicalein and ginsenoside are the most studied and have potential clinical applications. we summarized these four natural products in detail, with additional natural products outlined in **Table 2**.

**Table 2:** Effects of other natural products on autophagy and apoptosis in NSCLC.

Natural products	category	Cell Models/Animals Models	Targets/mechanisms	functions	references
Resveratrol	Phenols	A549, ASTC-a-1, H446 cell lines	Upregulate Bax, cleaved caspase-3; Downregulate Bcl-2, Bcl-2/Bax; Trigger Bak; Increase LC3-II/LC3-I, Beclin-1; Decrease p62 expression; Inhibit PI3K/Akt/c-Myc pathway; Activate NGFR-AMPK-mTOR Pathway	Induces apoptosis and autophagic death	(161–165)
Triptolide	Terpenoids	PC9, A549, H1395 cell lines	Inhibit activation of the IL-6/STAT3 axis; Decrease BCL-2, MCL-1; Increase cleaved caspase-3; Activate the calcium (Ca2+)/calmodulin-dependent protein kinase kinase β (CaMKKβ)/AMP-activated protein kinase (AMPK) pathway	Induce apoptosis	(166, 167)
Andrographolide	Terpenoids	H1975, H1299, H292, H1650, H460, BEAS-2B, H522, LLC cell lines; H1975-bearing mice	Downregulate SQSTM1 (sequestosome 1)/p62; Induce oxidative stress; Induce mitochondrial ROS generation and loss of MMP; Increase cleaved caspase−3, cleaved caspase−9 and PARP	Increase autophagy-induced cell death and promote apoptosis	(168, 169)
β-elemene	Terpenoids	H460, A549 cell lines	Induce caspase-3, -7, -9 activities; Cause cytochrome c release; Increase cleaved caspase-9; inhibit PI3K/Akt/mTOR/p70S6K1 pathway; Enhance the expression of Atg5-Atg12 conjugated protein; Increased the ratio of LC3-II to LC3-I; Downregulate Bcl-2, Survivin	Induce apoptosis and activate a protective autophagy	(170, 171)
Gambogic acid	Flavonoids	H441, SCLC, H446, H1688 cell lines; NCI−H441-bearing mice	Increase cleaved caspase-3, -8 and -9, Bax; Decrease Bcl-2; Upregulate Beclin 1 and conversion of LC3 I to LC3 II; Generated ROS	Induce apoptosis and autophagy	(172, 173)
Tanshinone IIA	Quinonoids	A549 cell line	decrease the MMP; Upregulate Bax/Bcl-2; Activate caspase-9, caspase-3, cytochrome c release and Bax translocation to the mitochondria	Induce apoptosis	(174, 175)
Emodin	anthraquinones	CH27, H460, A549, H1299 cell lines; A549-bearing mice	Increase cleaved caspase-3, cytochrome c of cytosolic fraction; Decrease CHOP; Trigger ER stress-mediated apoptosis	Induce apoptosis	(176, 177)

NGFR, the nerve growth factor receptor; MMP, mitochondrial membrane potential; CHOP, C/BEP homologous protein.

### 5.1 Dihydroartemisinin

Dihydroartemisinin (DHA), derived from artemisinin, is primarily known as a frontline antimalarial drug developed in China. Beyond its anti-malarial properties, an increasing body of research reports DHA’s anticancer activity against a range of cancers *in vivo* and *in vitro*. Its anticancer mechanisms encompass inhibition of proliferation, induction of apoptosis, reduction of tumor metastasis and angiogenesis, enhancement of immune function, and induction of autophagy and endoplasmic reticulum stress (178). Due to its established low toxicity and safety, DHA has been extensively studied in NSCLC treatment. **Table 3** summarizes DHA’s effects on autophagy and apoptosis in NSCLC.

**Table 3:** Effects of DHA on autophagy and apoptosis in NSCLC.

Author	Year	Cell Models/Animals Models	Targets/Mechanisms	Functions
Liu et al. (179)	2018	A549 cell line; A549-bearing mice	Upregulate GFP-LC3, HMGB1, LC3-II/LC3-I; Activate MAPK pathway	Induce autophagic cell death
Wu et al. (180)	2022	A549 cell line	Inhibit CIRBP; Inhibit the PINK1/Parkin pathway	Inhibit mitophagy to reduce radiation resistance
Zhang et al. (181)	2021	H1299, A549, LTEP-a-2, H23, H358 cell lines	Downregulate VDAC, ROS; Activate caspase 3	Induce ROS-dependent apoptosis
Yan et al. (182)	2018	H1975, HCC827, H1650, H3255, A549, H727, H1299, MV522 cell Lines; H1975-bearing mice	Downregulate Mcl-1 and Survivin; Upregulate Bim; Inhibit the JAK-STAT3 pathway	Induce Bax-dependent apoptosis
Liao et al. (183)	2014	A549 cell line	Upregulate Bax/Bcl-2; Activate caspase 3 and cytochrome-c	Induce apoptosis
Li et al. (184)	2021	A549, LLC cell lines; LLC-bearing mice	Downregulate Bcl-xl and Bcl-2; Inhibit mTOR/HIF-1α pathway	Induce apoptosis
Lai et al. (185)	2023	A549, HCC827, H1975 cell lines	Upregulate ROS, cleaved Caspase 3, LC3 and Beclin-1; Downregulate p62, Bcl-2	Synergy effect: upregulate autophagy and induce apoptosis

HMGB1, High mobility group box 1; VDAC, voltage-dependent anion channel; JAK-STAT3, Janus Kinase-signal transducer and activator of transcription 3; CIRBP, Cold-Inducible RNA Binding Protein; PINK1, putative protein kinase 1; LLC, Lewis lung cancer.

#### 5.1.1 Regulation of autophagy

Autophagy can either facilitate tumor survival or lead to autophagic cell death during tumor progression and therapy. This process is influenced by the levels of signaling molecules activated by therapeutic agents (186). Liu et al. discovered that DHA-37, a novel derivative of DHA, initiated MAPK signaling by upregulating High mobility group box 1 (HMGB1) in A549 cells. This activation led to excessive autophagic cell death, making autophagy a key factor in A549 cell mortality (179). Radiotherapy, a primary treatment for NSCLC, often encounters challenges with radiation resistance, a significant factor in residual and recurring cancer post-treatment. Research indicates that DHA can inhibit mitophagy, potentially decreasing radiation resistance and enhancing the therapeutic effect against NSCLC (180).

#### 5.1.2 Regulation of apoptosis

Numerous studies have established that DHA can significantly induce apoptosis in NSCLC cells *in vitro* and *in vivo*. Zhang et al. found that DHA exhibits anti-NSCLC activity by inducing ROS-dependent apoptosis (181). Another study highlighted that DHA effectively inhibited signal transducer and activator of transcription 3 (STAT3) phosphorylation. Inactivation of STAT3 led to reduced levels of Mcl-1 and Survivin, thereby exerting a synergistic anticancer effect and inducing apoptosis in NSCLC cells (182). Moreover, it has been reported that DHA induces apoptosis in the A549 lung cancer cell line, evidenced by an increased Bax/Bcl-2 ratio and elevated levels of active caspase-3 and cytochrome-c (183). Additionally, both *in vivo* and *in vitro* experiments demonstrate that DHA reduces the expression of the mTOR/HIF-1α signaling pathway and induces apoptosis in NSCLC (184).

#### 5.1.3 Regulation of autophagy and apoptosis

DHA plays a crucial role in enhancing anticancer effects and reversing drug resistance in NSCLC by regulating both autophagy and apoptosis. One study suggests that DHA treatment significantly increases the sensitivity of A549-GR cells to gefitinib by elevating apoptosis and upregulating autophagy, thereby effectively controlling tumor progression (185).

### 5.2 Curcumin

Curcumin, a polyphenol derived from the traditional Chinese medicine Curcuma longa, has shown anti-inflammatory, antioxidant, and anti-tumor properties (187). Its application in cancer treatment, particularly in NSCLC, has gained increasing interest. Research indicates that Curcumin can modulate various programmed cell death mechanisms, including autophagy, apoptosis, pyroptosis, and ferroptosis (188). **Table 4** summarizes Curcumin’s impact on autophagy and apoptosis in NSCLC.

**Table 4:** Effects of Curcumin on autophagy and apoptosis in NSCLC.

Author	Year	Cell Models/Animals Models	Targets/Mechanisms	Functions
Tang et al. (189)	2021	A549, H1299 cell lines; LLC-bearing mice	Upregulation Beclin-1, LC3; Downregulate P62	Induce autophagic ferroptosis
Zhou et al. (190)	2014	A549 cell line	Upregulation conversion of LC3-I to LC3-II	Induce autophagic cell death
Xu et al. (191)	2015	A549, H1299 cell lines	Increase cleaved caspase-3, cleaved caspase-9, [Ca^2+^]I, the phosphorylation of IP3R; Downregulate Bcl-2	Induced mitochondrial-dependent apoptosis
Jin et al. (192)	2015	H460, BEAS-2E, A549 cell lines	Increase caspase-3 activity; Upregulation microRNA-192-5p pathway; Suppress PI3K/Akt pathway	Induce apoptosis
Ye et al. (193)	2015	CH460, A427, A549, H1299 cell lines; H460, H1299-bearing mice	Increase caspase-3 activity; Upregulate microRNA -192-5p/215-XIAP pathway	Induce apoptosis
Wang et al. (194)	2017	A549, H1299 cell lines	Downregulate the phosphorylation levels of mTOR, ribosomal protein S6, phosphoinositide 3-kinase and Akt (protein kinase B); Suppress PI3K/Akt/mTOR pathway	Synergy effect: enhance autophagy and promote apoptosis
Liu et al. (195)	2018	A549 cell line	Increase LC3-II/LC3-I, Beclin-1, formation of autophagic vesicles; Decrease p62; Downregulate PI3K/Akt/mTOR pathway	Synergy effect: enhance autophagy and promote apoptosis

XIAP, X-linked inhibitor of apoptosis.

#### 5.2.1 Regulation of autophagy

Curcumin, a natural autophagy modulator, has the capacity to initiate autophagy in various cancers, including NSCLC (196). Tang et al. discovered that Curcumin caused mitochondrial membrane rupture and a decrease in mitochondrial cristae, enhanced autolysosome formation, increased the levels of Beclin-1 and LC3, reduced P62 levels, and induced ferroptosis by activating autophagy in NSCLC (189). Furthermore, a study revealed that hydrazinobenzoylcurcumin, a synthetic derivative of Curcumin, induces the conversion of LC3-I to LC3-II in A549 cells and enhances the fusion of autophagosomes with lysosomes, leading to autophagic cell death in cancer cells (190).

#### 5.2.2 Regulation of apoptosis

Curcumin has been demonstrated to trigger apoptosis in NSCLC through various mechanisms. Treatment with Curcumin decreased Bcl-2 expression and increased inositol 1,4,5-trisphosphate receptor (IP3R) phosphorylation in a concentration-dependent manner. This elevation led to increased intracellular free calcium levels, with calcium overload subsequently inducing mitochondrial-dependent apoptosis in lung cancer cells (191). Additional studies have shown that Curcumin induces apoptosis in human NSCLC cells by increasing caspase-3 activity and upregulating the miR-192-5p signaling pathway (192, 193).

#### 5.2.3 Regulation of autophagy and apoptosis

The PI3K/Akt/mTOR pathway is crucial for cell proliferation, apoptosis, autophagy, metabolism, and cell cycle progression, representing a key survival pathway often dysregulated in various human cancers (197). Multiple studies have demonstrated that Curcumin enhances autophagy and promotes apoptosis by downregulating the PI3K/Akt/mTOR pathway (194, 195).

### 5.3 Baicalein

Baicalein, a flavonoid extracted from the roots of *Scutellaria baicalensis*, has demonstrated substantial anti-tumor activity in recent *in vivo* and *in vitro* experiments, showing particular preventive and therapeutic effects on NSCLC (198). It has a wide anticancer spectrum, affects multiple cellular actions, and exhibits complex and diverse mechanisms, including the regulation of cell proliferation, metastasis, apoptosis, and autophagy (199). Baicalein is emerging as a novel anti-cancer agent for NSCLC treatment. **Table 5** summarizes the effects of baicalein on autophagy and apoptosis in NSCLC.

**Table 5:** Effects of baicalein on autophagy and apoptosis in NSCLC.

Author	Year	Cell Models/Animals Models	Targets/Mechanisms	Functions
Li et al. (200)	2021	H1299, A549 cell lines; H1299-bearing mice	Inhibit the kinase activity of MAP4K3; Activate TFEB; Regulate baicalein/MAP4K3/mTORC1/TFEB axis	Trigger TFEB-dependent autophagy to suppress tumors
Leung et al. (201)	2007	H460 cell line	Form vesicles and apoptotic body, DNA condensation and fragmentation; Downregulate Bcl-2 and caspase-3 proform levels; Increasing p53 and Bax; Inhibit 12-LOX	Induce apoptosis
Lee et al. (202)	2005	CH27 cell line	Downregulate Bcl-2 and caspase-3 proform levels	Induce apoptosis
Kim et al. (203)	2016	A549 cell line	Induce presence of condensed chromatin, apoptotic bodies and DNA fragmentations; Downregulate Bcl-xL and MMP; Promote ROS generation and AMPK activation; Activate caspase-3、8、9	Induce Caspase-dependent apoptosis
Yu et al. (204)	2017	A549 cell lines; A549-bearing mice	Downregulate the antiapoptotic proteins c-IAP1, c-IAP2, survivin, Bcl-Xl; Inhibit PI3K/Akt/NF-kB pathway	Induce apoptosis
Deng et al. (205)	2020	A549, H1299 cell lines; LLC-bearing mice	Activate AMPK pathway; Enhance fatal Drp1-mediated mitochondrial fission	Synergy effect: upregulate autophagy and induce apoptosis

12-LOX, 12-lipoxygenase; MMP, mitochondrial membrane potential; MAP4K3, mitogen-activated protein kinase kinase kinase kinase 3; TFEB, transcription factor EB.

#### 5.3.1 Regulation of autophagy

mitogen-activated protein kinase kinase kinase kinase 3 (MAP4K3) is a key regulator of mammalian cell growth and autophagy. Baicalein can directly bind to MAP4K3, leading to its inactivation. The degradation of MAP4K3 triggers transcription factor EB (TFEB)-dependent autophagy, causing arrest in lung cancer cell proliferation and effectively inhibiting tumor growth. Thus, baicalein regulates autophagy in NSCLC cells via the MAP4K3/mTORC1/TFEB axis (200).

#### 5.3.2 Regulation of apoptosis

Bcl-2 and caspase-3 are crucial in determining cellular apoptosis. Studies indicate that baicalein induces apoptosis in NSCLC cells by downregulating Bcl-2 and Caspase-3 proform (201, 202). Another research found that baicalein’s apoptotic activity might be linked to a caspase-dependent cascade reaction (203). Additionally, baicalein promotes apoptosis in tumor cells by inhibiting NF-kB-mediated anti-apoptotic proteins, including c-IAP1, c-IAP2, survivin, and Bcl-xL, via the PI3K/Akt/NF-kB pathway. This inhibition significantly enhances NSCLC chemosensitivity to cisplatin (204).

#### 5.3.3 Regulation of autophagy and apoptosis

Recent research indicates that baicalein modulates apoptosis and autophagy in NSCLC via the AMPK/Drp1/mitochondrial fission axis. Baicalein activates the AMPK pathway and intensifies fatal dynamin-related protein 1 (Drp1)-mediated mitochondrial fission. This process leads to the loss of mitochondrial membrane potential and the release of cytochrome c and apoptosis-inducing factor from mitochondria to cytoplasm. Such actions stimulate the combined anticancer effects of autophagy and apoptosis (205).

### 5.4 Ginsenoside

Ginseng is highly valued as both a medicinal and food ingredient, with ginsenosides being its most crucial constituents. Extensive research has focused on ginsenosides in cancer chemoprevention and therapy, identifying about 200 different ginsenosides in ginseng. Notably, ginsenosides Rk1, Rk3, Rg3, Rh2, and CK have been recognized for their potent anticancer effects and are currently at the forefront of scientific investigation (206). Their mechanisms are primarily associated with cell cycle arrest, apoptosis, proliferation, invasion, metastasis, angiogenesis, autophagy, and immune response (207). **Table 6** summarizes the effects of ginsenosides on autophagy and apoptosis in NSCLC.

**Table 6:** Effects of ginsenoside on autophagy and apoptosis in NSCLC.

Author	Year	Ginsenoside Extract	Cell Models/Animals Models	Targets/Mechanisms	Functions
Zhao et al. (208)	2019	Total ginsenosides extract	A549, PC-9 cell lines	Upregulate LC3 II; Activate the ATF4-CHOP-AKT1-mTOR axis	Induce autophagic cell death
Hu et al. (209)	2020	Ginsenoside Rk1	A549, PC9 cell lines; A549-bearing mice	Increase Bax, cleaved caspase-3, -8, -9, PARP; Decrease Bcl-2, PD-L1; Block NF-κB pathway	Induce apoptosis
Duan et al. (210)	2017	Ginsenoside Rk3	H46, A549 cell lines; H460-bearing mice	Activate caspase-3, -8, -9; Decrease Bcl-2; Increase Bax expression and causes cytochrome c release	Induce apoptosis
Xie et al. (211)	2017	Ginsenoside Rg3	A549, H23 cell lines; A549, H23-bearing mice	Inhibit PI3K/Akt pathway	Induce apoptosis
Hwang et al. (212)	2022	Red ginseng extract Rg3	A549, H460 cell lines	Increased PINK1, Parkin protein (Ser65) phosphorylation; Overproduce ROS	Lead to apoptosis and mitophagy
Li et al. (213)	2019	Ginsenoside Compound K	A549, H1975 cell lines	Regulate AMPK/mTOR pathway; Activate JNK pathway; Increase LC3 II and Beclin-1 levels; Decrease p62 level	Promoting effect: induce autophagy-mediated apoptosis
Chen et al. (214)	2020	Ginsenoside Rh2	A549 cell line	Repress EGFR-PI3K-AKT Pathway; Eliminate ROS; Decrease PD-L1 expression	Antagonism effect: enhance apoptosis by repressing autophagy

#### 5.4.1 Regulation of autophagy

Autophagic cell death, classified as a type II programmed death, is an effective strategy for inducing tumor cell death in lung cancer. One study demonstrated that total ginsenosides extract (TGS) could enhance autophagy flux and induce autophagic cell death in NSCLC cells. This is achieved by activating endoplasmic reticulum stress, with further investigations indicating the involvement of the Activating Transcription Factor 4 (ATF4)- C/EBP-homologous protein (CHOP)-AKT1-mTOR axis in this process (208).

#### 5.4.2 Regulation of apoptosis

Numerous studies have validated the role of ginsenosides in inducing apoptosis in NSCLC. A study revealed that ginsenoside Rk1 increases the protein expression of Bax, cleaved caspase-3, -8, -9, and poly ADP-ribose polymerase (PARP), while decreasing Bcl-2 expression. It also inhibits the NF-KB signaling pathway and reduces the high expression of PD-L1, thus significantly promoting apoptosis in NSCLC cells (209). Another research showed that ginsenoside Rk3 triggers apoptosis in H460 and A549 cells through death receptor-mediated, mitochondria-dependent pathways (210). Xie et al. reported that ginsenoside Rg3 significantly reduces cell viability and induces apoptosis by inhibiting the PI3K/Akt signaling pathway in A549 and H23 cells both *in vitro* and *in vivo* (211).

#### 5.4.3 Regulation of autophagy and apoptosis

Red ginseng, a steamed form of ginseng, has been shown to promote lung cancer cell apoptosis and mitophagy. This is achieved through the activation of ROS and the PINK1-Parkin signaling pathway, as reported in a study focusing on Rg3-enriched red ginseng extract (212). Another study found that a metabolite of ginsenoside, Compound K (C-K), induces autophagy-mediated apoptosis in A549 and H1975 cells by regulating the AMPK/mTOR and JNK signaling pathways (213). Platinum-based drugs are first-line treatments for NSCLC, but cisplatin-induced productive autophagy can lead to drug resistance. Reversing this resistance is thus a key strategy in NSCLC treatment. Chen et al. discovered that ginsenoside Rh2 enhances cisplatin-induced apoptosis in A549 cells by repressing autophagy, significantly hindering NSCLC progression (214).

## 6 Problems and prospects

This paper initially introduced the mechanisms of autophagy and apoptosis, analyzed the crosstalk molecules between them, and then summarized their roles in NSCLC. Finally, we reviewed natural products that have been intensively studied against NSCLC targeting autophagy and apoptosis, and summarized in detail the four most retrieved representative drugs. Previous results and studies indicate that in NSCLC, natural products can regulate many target proteins on autophagy and apoptosis signaling pathways, inducing autophagic death and apoptosis in tumor cells. They also stimulate the synergistic and promotional effects of autophagy and apoptosis to co-induce tumor cell death. Additionally, natural products enhance apoptosis in tumor cells while reversing drug resistance by downregulating autophagy.

In conclusion, natural products effectively and reasonably regulate autophagy and apoptosis in NSCLC, exhibiting significant potential as a novel therapeutic strategy. Their sources are diverse, offering multiple targets, high safety, and effectiveness as anticancer agents. The data indicate that numerous natural products have been discovered through fundamental and preclinical studies for treatment of NSCLC. Despite this, there has not been a corresponding increase in the number of drugs used in the clinical setting, and possible reasons for this include the following six areas. Firstly, the discovery of new pharmacologically active prototypes in natural products is slow. Researchers should employ modern screening techniques to explore, extract, and identify natural products, assess their efficacy, safety, and bioavailability, and standardize promising anticancer natural products. Secondly, repositioning existing drugs for new applications is a growing trend in drug research and development, offering cost and time savings and resource optimization. Yet, the exploitation of new applications and anticancer domains for natural products is currently limited. Thirdly, Single natural product therapies have limited efficacy in treating and preventing cancer. literature on the combination of natural products is sparse. Researchers should focus on multi-molecule combination therapies using natural products, studying their synergistic anticancer effects. Additionally, natural products can serve as chemosensitizers, enhancing the effects of various chemotherapeutic agents and radiotherapy. And fourth, natural products exhibit side effects akin to other substances. For example, the side effects of camptothecin include bone marrow suppression, nausea, vomiting, stomatitis, abdominal pain, fatigue, diarrhea, peripheral neuropathy and hair loss. Therefore, the development of relevant technologies to aid in selecting the optimal therapeutic dose, design rational dosage forms, and alleviating their side effects and toxicity to the host is critical. Fifth, while numerous studies on natural products for cancer treatment have been documented, the majority have focused on *in vitro* experiments, with limited *in vivo* data and therefore restricted potential efficacy in human subjects. Researchers should carry out relevant *in vivo* experimental studies and gradually advance toward clinic trial. At last, Natural product active ingredients often face challenges like poor stability, low water solubility, rapid decomposition and metabolism, and low bioavailability, which can limit their clinical applications.

Excitingly, the advancement of novel drug delivery systems utilizing material technology and nanotechnology heralds a promising new research arena for anticancer drugs. The use of nanoscale materials such as nanoparticles, micelles, liposomes, microspheres, and microcapsules to physically or chemically encapsulate or link natural products and their derivatives for drug delivery presents a potential solution to overcome the shortcomings of natural products. Therapeutic strategies that integrate the benefits of modern delivery technologies with traditional medicines are poised to reshape the landscape of anticancer drugs in the coming decade.

## Author contributions

PQ: Writing – original draft, Conceptualization, Writing – review & editing. QL: Writing – original draft, Conceptualization, Data curation. QZ: Data curation, Writing – original draft. RD: Data curation, Writing – original draft. YQ: Writing – review & editing, Conceptualization.
